# Prognostic value of histological and biological markers in pharyngeal squamous cell carcinoma – a reply

**DOI:** 10.1054/bjoc.1999.1189

**Published:** 2000-04-03

**Authors:** M Guerry, L Vabre, M Talbot, G Mamelle, A M Leridant, C Hill, J Bosq, B Luboinski, F Janot

**Affiliations:** Department de Chirugie Cervico-faciale, Institut Gustave Roussy, 39 Rue Camille, Desmoulins, 94805, France


essential for two reasons. Firstly, patients with positive neck nodes have a higher risk of developing DM as have those with negative neck nodes (Leemans et al, 1993; Mamelle et al, 1994). Secondly, a heterogeneity between the tumour cell populations of the primary tumour site and the neck nodes is possible. Therefore an analysis of the distribution of markers in the cervical nodes is essential. Consequently, the results are questionable at this point in time.

A case-control study in which only patients with negative neck nodes are selected will define which immunohistochemical markers are related to DM.

*F Carinci[1] and PF Carls[2]*
*[1]Assistant Professor Maxillofacial Surgery, Ferrara University, Italy; [2]Consultant Oral & Maxillofacial Surgeon, John Radcliffe Hospital, Oxford OX3 9DU, UK*

### REFERENCES

Guerry M, Vabre L, Talbot M, et al (1998) Prognostic value of histological and biological markers in pharyngeal squamous cell carcinoma: a case control study. *Br J Cancer* **77**: 1932–1936

Leemans C, Tiwari R, Nauta J, et al (1993) Regional lymph node involvement and its significance in the development of distant metastases in head and neck carcinoma. *Cancer* **71**: 452–456

Mamelle G, Pampurik J, Luboinski B, et al (1994) Lymph node prognostic factors in head and neck squamous cell carcinomas. *Am J Surg* **168**: 494–498

DOI: 10.1054/ bjoc.1999.1189, available online at http://www.idealibrary.com on IDEAL®

### Prognostic value of histological and biological markers in pharyngeal squamous cell carcinoma – a reply

**Sir**,
As explained in our paper, the aim of our work was to study if histological or biological factors, determined on initial biopsy before treatment, could provide new prognostic information. It is a clinical question, because it can help the clinician to choose the initial treatment of the patient. For example, in the group of patients with a higher risk to develop distant metastases, the clinician could choose to begin with a general treatment (neo-adjuvant chemotherapy for example) before the loco-regional treatment. For that reason we focused on histo-biological factors which can be determined before treatment.

The idea of studying immunohistochemical markers on neck nodes is interesting, but it is a different question: it can only be determined after surgery.

Nevertheless, the suggestion of Drs Carinci and Carls is important. We are currently performing new studies so as to better understand our results. We have seen that the expression of c-*erb*-B2 can be present in non-transformed mucosa and disappear in adjacent transformed mucosa (discussion of our paper). It will be interesting to see if, when expressed in tumour, it can also disappear in involved lymph nodes.

Thank you for your interest in our work.

*M Guerry, L Vabre, M Talbot, G Mamelle, AM Leridant, C Hill, J Bosq, B Luboinski and F Janot*
*Department de Chirugie Cervico-faciale, Institut Gustave Roussy, 39 Rue Camille, Desmoulins 94805, France*

 *© 2000 Cancer Research Campaign*

